# Investigating the Reduction/Oxidation Reversibility of Graphene Oxide for Photocatalytic Applications

**DOI:** 10.3390/molecules28114344

**Published:** 2023-05-25

**Authors:** László Péter Bakos, Marcell Bohus, Imre Miklós Szilágyi

**Affiliations:** Department of Inorganic and Analytical Chemistry, Budapest University of Technology and Economics, Szent Gellért tér 4, H-1111 Budapest, Hungaryszilagyi.imre.miklos@vbk.bme.hu (I.M.S.)

**Keywords:** graphene oxide, reduced graphene oxide, photocatalysis

## Abstract

The aim of the study was to analyze the reversibility of the cycle of graphene oxide (GO), reduced GO, and GO obtained by consecutive reoxidation of reduced GO. Accordingly, GO was heated in three different atmospheres (oxidizing, inert, and reducing, i.e., air, nitrogen, and argon/hydrogen mixture, respectively) at 400 °C to obtain reduced GO with varying composition. The bare GO and the RGO samples were oxidized or reoxidized with HNO_3_. The thermal properties, composition, bonds, and structure of the samples were investigated with TG/DTA, EDX, Raman spectroscopy, and XRD. Their photocatalytic activity was tested by decomposing methyl orange dye under UV light irradiation.

## 1. Introduction

Carbon-based nanostructures (graphene, graphene-oxide, carbon aerogels, carbon nanospheres, carbon nanotubes, fullerenes, nanodiamonds, etc.) have been widely investigated materials in the recent decades because of their many outstanding properties, such as high specific surface area (SSA), tunable properties such as thermal and electrical conductivity, catalytic activity, thermal stability etc., achieved by controlling their composition and functional groups. For example, to these statements Lu et al. prepared graphene-like nanosheets from biomass with a maximum surface area of 1015.0 m^2^/g [1]. Akhavan created graphene nanomesh with the utilization of vertically ZnO nanorods, which showed a p-type semiconducting behavior [2]. Selective catalytic activity of graphene oxide was reported by Sedrpoushan et al. They used graphene oxide nanoparticles to oxidize aromatic aldehydes and alcohols in the presence of H_2_O_2_ [3]. Graphene nanomaterials are excellent candidates for fire retardants due to their high thermal stability, conductivity, and customizability. Ma et al. studied graphene quantum dots as fire retardants in polystyrene, while Chen et al. experimented with functionalized graphene and boron nitride [4,5]. Graphene nanomaterials are also used for their high thermal conductivity in heat exchangers and solar collectors [6,7]. These examples show the wide usability of graphene in different fields of application.

Carbon nanostructures, especially graphene oxide (GO) and reduced GO (RGO), are often used as substrates for photocatalysts. Commonly for TiO_2_ [8,9,10], CuO [11], and ZnO [12], they can enhance their activity by increasing the surface area of the photocatalyst, sensitizing to visible light, and inhibiting the recombination of the photogenerated electrons and holes through their electron acceptor nature [13,14,15]. Additionally, rarer elements, such as bismuth, can also be combined with graphene oxide for enhanced abilities [16]. Moreover, several nanostructured carbons, such as graphene oxide, carbon aerogels [17], nanospheres [18], dots [19], and nanotubes [20], can possess photocatalytic properties by themselves, which are not widely investigated [21]. It is worth to mention that there are some special combinations of the mentioned photocatalysts, such as composites created by the utilization of atomic layer deposition [22,23] or the stimulation of neural cells on graphene oxide nanomesh [2,24].

Previous results suggest that the functional groups (mostly oxygen containing ones) present on the surface of the carbon materials are crucial in the process of photon absorption and electron-hole generation for the photoreaction [25,26]. The number of functional groups and the overall composition as well change considerably when GO is transformed into RGO. However, it was unclear whether this process could be reversed and RGO could be transformed back into GO, what properties the reobtained GO might have, and how different the bare and regained GO samples might be [27,28,29]. Raja et al. made a comparative study between GO and RGO flakes regarding their photochemical and electrochemical properties. They reported that their band gap is 3.61 and 3.38 eV, respectively, which makes them suitable photocatalysts with UV irradiation. This is confirmed by the photocatalytic decomposition of ciprofloxacin. Cyclic voltammetry (CV) measurements showed that the specific capacitance of RGO improved to 236 F/g at 1 A/g current density [30]. With heat treatment, the specific capacitance of GO can also be improved. CV results show that the specific capacitance is increased to 164.9 F/g with heat treatment in air at 200 °C [31]. Research suggests that there is a correlation between the electrical properties and the SSA of GO, which might correlate with the degree of oxidation. The SSA can vary largely depending on the stacking of layers from 500–700 m^2^/g (graphene nanoplatelets) to 2630 m^2^/g (single graphene sheet) [32,33].

Based on these, in this work, we used a bare graphene oxide as starting material and reduced it into RGO with heat treatments in three different atmospheres (oxidizing, inert, and reducing, i.e., air, nitrogen, and argon/hydrogen mixture, respectively) at 400 °C. Consecutively, the RGO samples were reoxidized by reaction with HNO_3_. As reference, the bare GO was also treated by HNO_3_. The thermal reduction was followed by TG/DTA, and the composition, bonds, and structure of the samples were characterized with SEM, EDX, Raman spectroscopy, and XRD techniques. Their photocatalytic activity was tested in decomposing methyl orange dye under UV light irradiation, and the effect of the GO reduction and reoxidation was investigated on the photocatalytic model reaction.

## 2. Results and Discussion

### 2.1. Thermal Analysis

**Figure 1:**
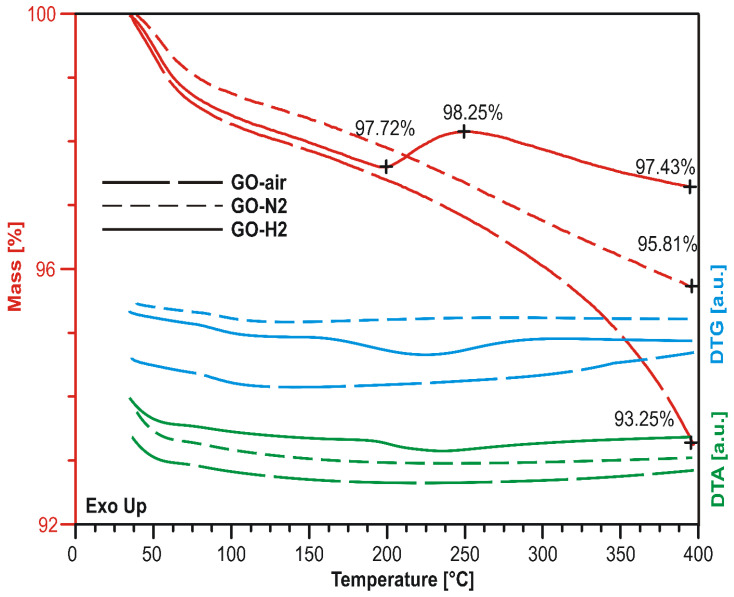
TG/DTA analysis of the GO in air, nitrogen, and argon/hydrogen mixture.

The TG/DTA results in Figure 1 show the thermal behavior of the bare graphene oxide in different atmospheres. In all cases, the adsorbed water left at first, which already started at 50 °C. After further heating, in air and nitrogen atmospheres, the graphene oxide decomposed steadily. This went to a greater extent, i.e., up to 6.75% mass decrease in air, as oxygen also oxidized the carbon backbone and the functional groups, while in nitrogen only the thermal cleavage of the functional groups happened without further decomposition and the final mass loss was only 4.19%. In argon/hydrogen atmosphere, the mass started to increase above 200 °C, along with an endothermic peak on the DTA curve, suggesting the hydrogenation of the aromatic rings, with a maximum increase in mass of 0.53%. This process stopped at around 260 °C, and the mass began to decrease, as the decomposition continued; however, the mass loss in the end was the smallest, i.e., only 2.67% [34,35,36].

### 2.2. Energy Dispersive X-ray Microanalysis

**Figure 2:**
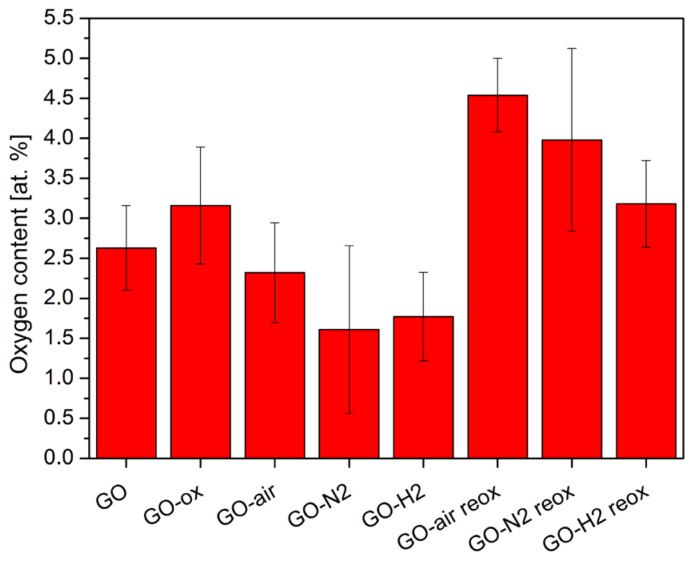
Oxygen content of the samples from EDX measurements.

According to the EDX measurements in Figure 2, oxidizing the bare GO sample only resulted in a slight increase in the oxygen amount. In contrast, the amount of oxygen decreased significantly during the heat treatments. When GO was annealed in air, parallel to the mass loss new oxygen containing functional groups could have formed as well. Therefore, the decrease of oxygen content was the lowest, while in the case of heating in nitrogen and hydrogen the oxygen amount reduced more significantly.

After reoxidizing the heat-treated samples, the oxygen content increased. The highest O amount was achieved for the GO sample annealed in air and then reoxidized (GO-air reox sample), because the heat treatment in air already created oxygen functionalities, and the reaction with HNO_3_ increased their amount further. In the case of the sample GO-N2 reox, less oxygen containing functional groups formed than for the GO-air reox sample, but considerably more compared to the GO-H2 reox sample, where the increase was the smallest. The reason for the lowest reoxidation ability of the GO-H2 reox sample may be the presence of the hydrogenated aromatic rings formed during annealing in H_2_/Ar, which inhibited the oxidation process in nitric acid. No nitrogen impurity was detectable originating from the HNO_3_ used for the reactions in the case of the oxidized samples.

### 2.3. Raman and FTIR Spectroscopy

**Figure 3:**
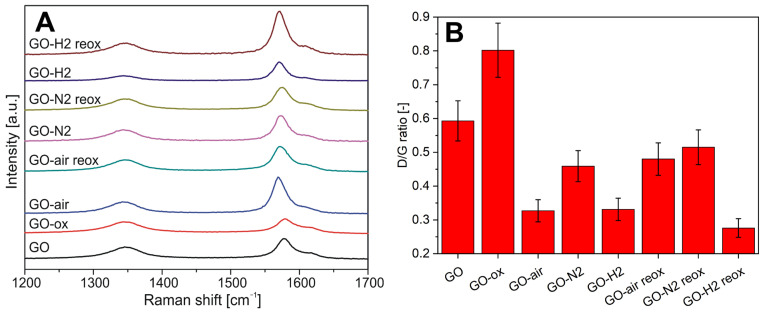
Raman spectra of the samples (**A**) and the ratio of the D and G peaks of the carbon (**B**).

On the Raman spectra in Figure 3A, the D (disordered, at ~1350 cm^−1^), G (graphitic, at ~1570 cm^−1^), and the D’ (from surface impurities, at ~1620 cm^−1^) peaks of carbon are visible for each sample. Figure 3B shows the ratio of the intensity of the D and G peaks (ID/IG), which indicates the level of graphitization [37].

For the GO-ox sample (GO oxidized without annealing), the ratio increased because of the new functional groups increased the disorder. Just the opposite, the thermal treatments reduced the ID/IG ratio. Its value was similar for the samples annealed in air and in argon/hydrogen, while it was less in nitrogen. This indicates that the presence of oxygen and hydrogen during the treatments helped the regularization of the graphene structure.

After reoxidation, the ID/IG ratio increased for samples preheated in air and nitrogen via the as-formed many new functional groups, and the increase in air was more notable. In the case of the sample preheated in hydrogen, an opposite process could be observed, i.e., the ID/IG ratio decreased even more. This indicates, along with the EDX results, that the hydrogenated aromatic ring decreases the effectiveness of the oxidation process with HNO_3_ [38].

FTIR spectra for sample GO (Figure S3) show the characteristic vibrations observed in graphene oxide [39,40].

**Figure 4:**
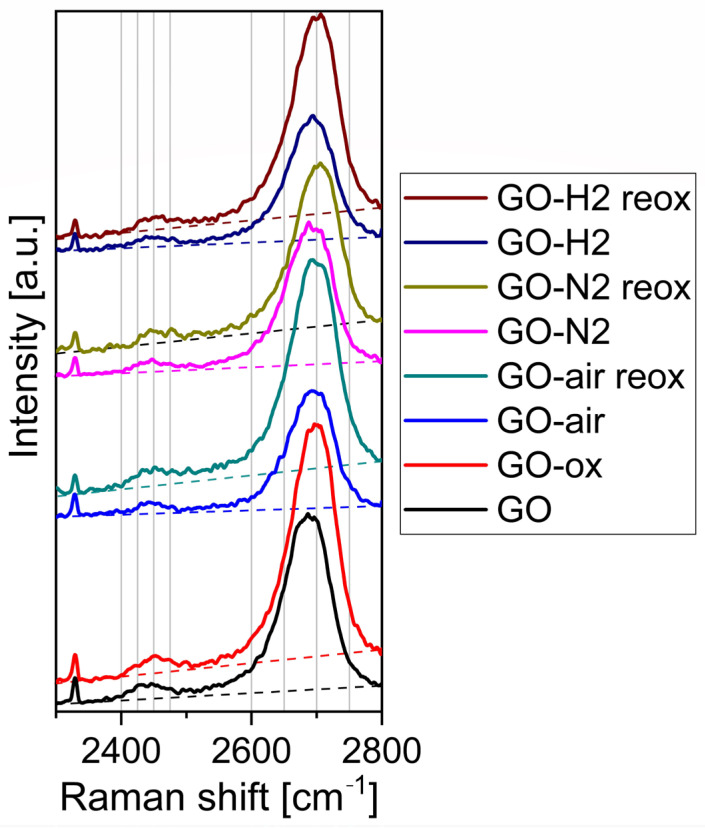
G* and 2D Raman modes of graphene oxide samples.

Figure 4 shows the Raman spectra from 2300 to 2800 cm^−1^. The bands around 2450 and 2700 cm^−1^ are assigned to G* and 2D (G′) Raman modes, respectively. The analysis of the spectra shows that the heat treatment does not affect the bands shape in neither atmosphere. On the contrary, with the acid treatment, the bands are shifted to the right, to higher wavenumbers. According to the manufacturer, the original GO sheets contain ten to fifteen layers, which then remains during heat treatment. The shift in the spectra indicates that the acid treatment increases the layers number [37,41]. At 2330 cm^−1^ a sharp peak is present which is not connected to the samples as it is caused by the N_2_ present in the atmospheric air.

### 2.4. XRD

**Figure 5:**
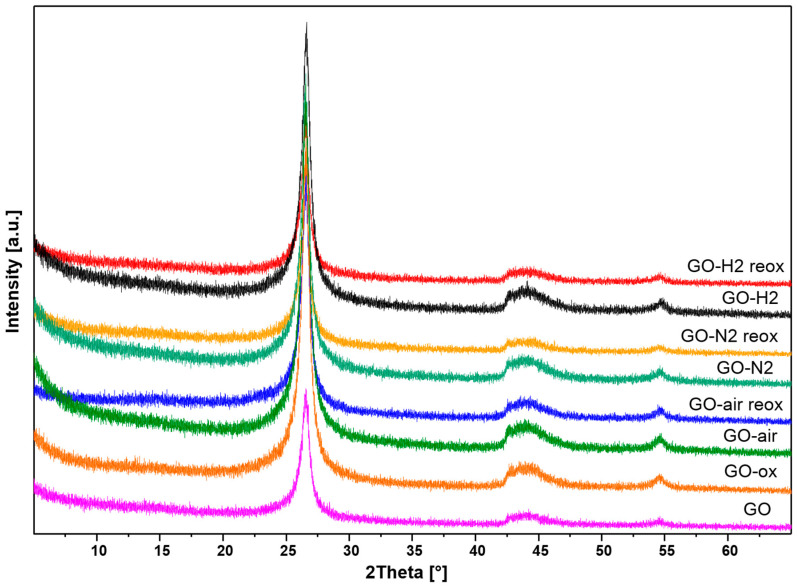
X-ray diffractograms of the samples.

According to the X-ray diffractograms in Figure 5, the peaks present belong to the Miller indices of 002 at 26°, 100 at 42°, 101 at 44°, and 004 at 54° of the graphene in the case of all samples. The characteristic graphene oxide peak around 12° is not visible, because the samples are only oxidized to a relatively small extent, even after the reaction with nitric acid [42,43]. Almost no difference was shown between the X-ray patterns, the calculated 002 interlayer distance was about 0.335 nm for all samples, which indicates that mostly the surface functional groups of the samples were affected by the utilized thermal reducing and chemical oxidizing treatments.

### 2.5. Morphology

**Figure 6:**
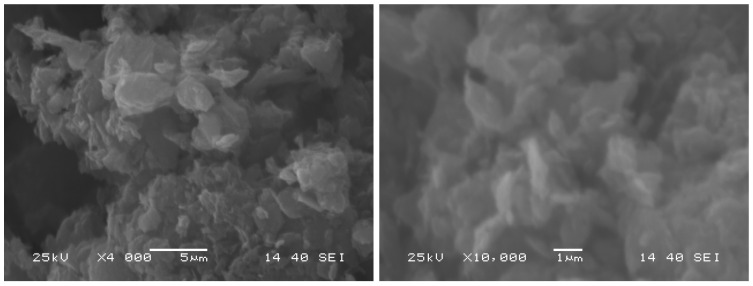
SEM images of graphene oxide.

The SEM pictures in Figure 6 show that, even though the particle size is on the nanometer scale according to the manufacturer, with a thickness of 15–20 sheets, they tend to create larger clusters which can reach several μm in one dimension. These clusters need to be split up, so the dye molecules can reach the surface. One effective way, to create a mostly stable dispersion of graphene oxide in water is to utilize sonication to the dispersions. We observed that graphene particles that were reoxidized were easier to disperse. The graphene oxide from the manufacturer is 4–6% edge oxidized and has less hydrophobe than bare graphene, but, to create a stable suspension in water, sonication is required. Temporal stability of this suspension can be reached, but it will sediment over time. Aggregation was not observed. With heat treatment, the decreased oxygen content caused lower stability, while increasing the oxygen content with reoxidation resulted in longer suspension stability. This could be due to the increased number of surface functional groups, containing oxygen that makes the surface of the particles more hydrophilic.

### 2.6. Photocatalysis

**Figure 7:**
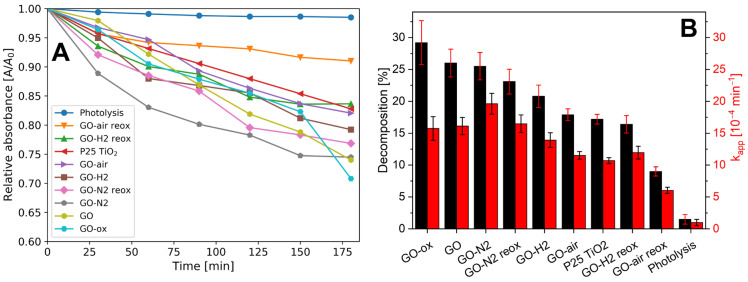
Measurement of the photocatalytic activity (**A**) and the decomposed amount of methyl orange and apparent reaction rate constants for the samples (**B**).

The results of the photocatalytic model reaction are shown in Figure 7A. The assumed pseudo-first order kinetics with Langmuir–Hinshelwood mechanism was adequate, as the R^2^ of the fitting was above 0.9 in all cases, the calculated apparent rate constants and the amount of decomposed methyl orange are shown in Figure 7B.

All samples possessed photocatalytic activity. All of them were better than the reference P25 TiO_2_, except for GO-air and GO-H2. The reoxidation after heat treatment decreased the activity in all cases. In contrast, for bare GO the oxidation was beneficial in respect to photocatalytic activity. On one hand, the reason for the activity decrease can be explained by that, after heat treatment, the loss of surface functional groups made the samples more hydrophobic and harder to reach by the dye molecules. On the other hand, the reoxidation inhibited the activity further, suggesting that just adding new functional groups on the heat-treated graphene oxides is not enough to make them better at photocatalysis. Additionally, the shift in the 2D Raman modes indicate the increase in graphene layer number thus the surface area of the acid-treated samples are lower than the original and annealed ones. This suggests that heat treatment makes the samples more capable for reoxidation but also lowers the catalytic activity.

## 3. Materials and Methods

### 3.1. Preparation of Samples

Graphene oxide (GO) (powder, 15–20 sheets, 4–10% edge-oxidized) and cc. nitric acid was obtained from Sigma-Aldrich, St. Louis, MO, USA. Heat treatments were performed in a TG/DTA machine (TA Instruments SDT 2960) with 7 mg GO powder, at a heating rate of 10 °C/min until 400 °C then cooldown to room temperature in 60 min using Pt crucible. Three different atmospheres were used, air as oxidizing, nitrogen as inert, and 95% argon/5% hydrogen as reducing, and the gas flow was 130 cm^3^/min in all cases.

**Figure 8:**
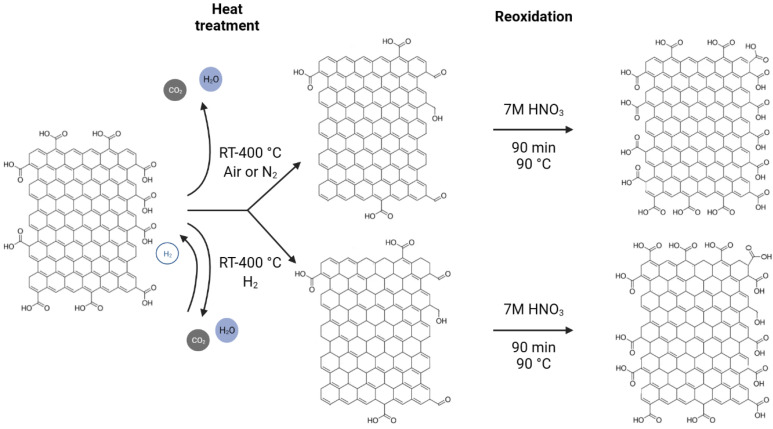
Schematic diagram of the reduction/reoxidation of graphene oxide.

Oxidation was made by putting the GO and the heat-treated samples into 7 M HNO_3_ solutions and stirring them at 90 °C for 1.5 h, then centrifuging and washing the suspensions with ion-exchanged water until the pH became neutral [44]. The schematic diagram of these processes is shown in Figure 8.

### 3.2. Characterization Methods

Thermogravimetry/differential thermal analysis (TG/DTA) measurements were made in parallel with the heat treatments (see previous section). Energy dispersive X-ray analysis (EDX) measurements and the acquisition of scanning electron microscopy (SEM) images were done on a JEOL 5500 scanning electron microscope, and the composition from EDX spectra was averaged from three areas for each sample. A Jobin Yvon Labram Raman spectrometer was used with a green Nd-YAG laser (532 nm) to get the Raman data. The FTIR spectra were measured on a Perkin Elmer System 200 FT-IR device using KBr pastilles. X-ray diffractograms (XRD) were made on a Panalytical X’Pert Pro MPD instrument with Cu Kα radiation.

**Figure 9:**
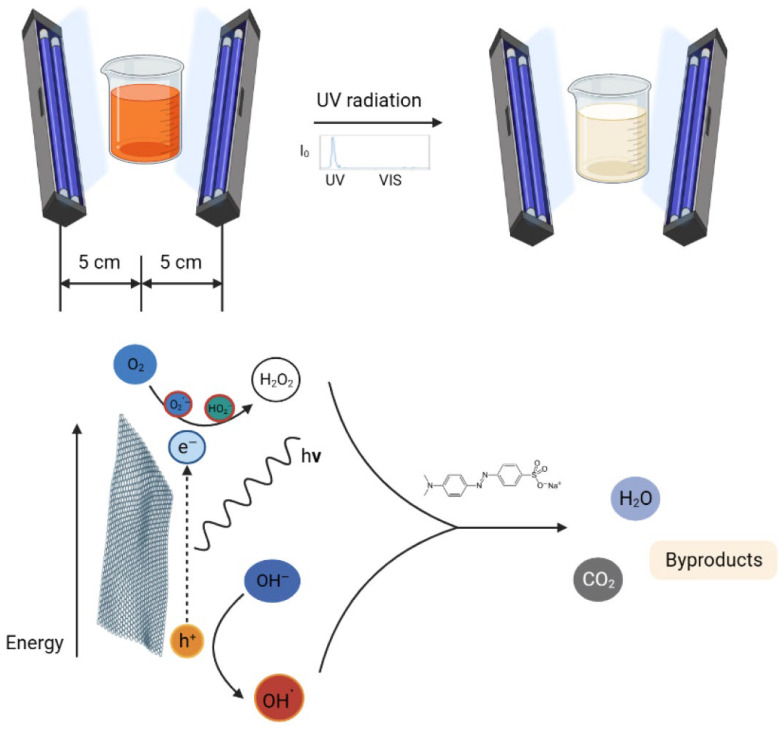
Schematic diagram of the photocatalytic decomposition of methyl orange.

To test the photocatalytic activity, a 1 mg sample was put into a quartz cuvette with 3 cm^3^ aqueous methyl orange solution with a concentration of 4 × 10^−5^ M and left in the dark for one day for the adsorption equilibrium to occur due to the high SSA of GO. Control measurements showed that the adsorption equilibrium is reached within half an hour, shown in Figure S1. For reference, P25 TiO_2_ (Sigma-Aldrich) was utilized because it is a commonly used and highly active photocatalyst. After one day adsorption in the dark, the cuvettes were put between two parallel Osram 18 W Blacklight UV lamps (spectrum: Figure S2), 5 cm from each, and they were irradiated for 3 h. The decomposition of the dye was followed by measuring the absorbance of the most intensive peak at 464 nm of the methyl orange with a Jasco V-550 spectrophotometer in every half hour. A schematic diagram of the photocatalytic decomposition is shown in Figure 9.

## 4. Conclusions

In this study, we have shown the oxygen functionalities can be removed and readded with thermal treatment and subsequent oxidation. When GO was annealed, it lost considerable amount of its functional groups. The highest mass loss was observed in air, as oxygen also oxidized the carbon backbone and the functional groups, while in nitrogen only the thermal cleavage of the functional groups happened without further decomposition. The smallest mass loss happened during annealing in the reducing atmosphere, where parallel to decomposition hydrogenation also occurred above 200 °C in argon/hydrogen atmosphere, as it was indicated by the TG/DTA measurements. Corroborating the thermal analysis data, EDX also confirmed that the annealing reduced the oxygen content of the samples. In the case of the chemical oxidation, the oxygen content increased for almost all samples. According to Raman measurements, the heat treatments helped the regularization of the graphene structure. In contrast, the chemical oxidation of the bare GO and the annealed samples increased the disorder, as it was seen in the increase in the D and G peak ratio. The graphene layers number increased during acid treatment according to the 2D Raman modes. Based on both Raman and EDX data, the hydrogenated aromatic rings formed during annealing in reducing atmosphere and decreased the effectiveness of the oxidation process with HNO_3_. In the XRD diffractograms, no significant difference was shown between the various samples, indicating that the thermal and chemical treatments only affected the surface of the samples. All samples showed photocatalytic activity, and most of them were even better than the reference P25 TiO_2_. The photocatalytic activity of the bare graphene oxide can be further increased by the HNO_3_ pretreatment, while for the samples the chemical reoxidation after heat treatments was not beneficial for the photocatalysis, independently of the atmosphere.

## Data Availability

The data presented in this study are available on request from the corresponding authors.

## Supplementary Materials

The following supporting information can be downloaded at: https://www.mdpi.com/article/10.3390/molecules28114344/s1, Figure S1: Reaching the adsorption equilibrium in dark using methyl orange and GO; Figure S2: Spectrum of the UV lamp used for the photocatalytic experiments; Figure S3: FTIR spectra for sample GO.
