# Supplementary material for: Investigating the Reduction/Oxidation Reversibility of Graphene Oxide for Photocatalytic Applications
Source: Molecules. 2023 May 25;28(11):4344. doi: 10.3390/molecules28114344 (PMC10254559; doi:10.3390/molecules28114344)
Supplement: Supplementary file 1 [file molecules-28-04344-s001.zip › molecules-2273980-supplementary.pdf]

Supporting information for

Investigating the reduction/oxidation reversibility of graphene oxide for photocatalytic applications

László Péter Bakos, Marcell Bohus * and Imre Miklós Szilágy

Department of Inorganic and Analytical Chemistry, Budapest University of Technology and Economics, Szent Gellért tér 4, H-1111 Budapest, Hungary;

szilagyi.imre.miklos@vbk.bme.hu (I.M.S.)

* Correspondence: bohusm@edu.bme.hu

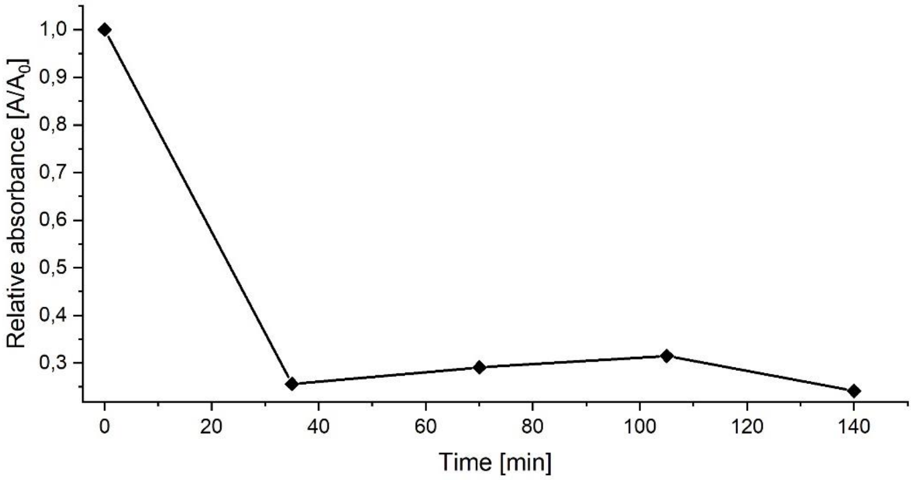

Figure S1. Reaching the adsorption equilibrium in dark using methyl-orange and GO

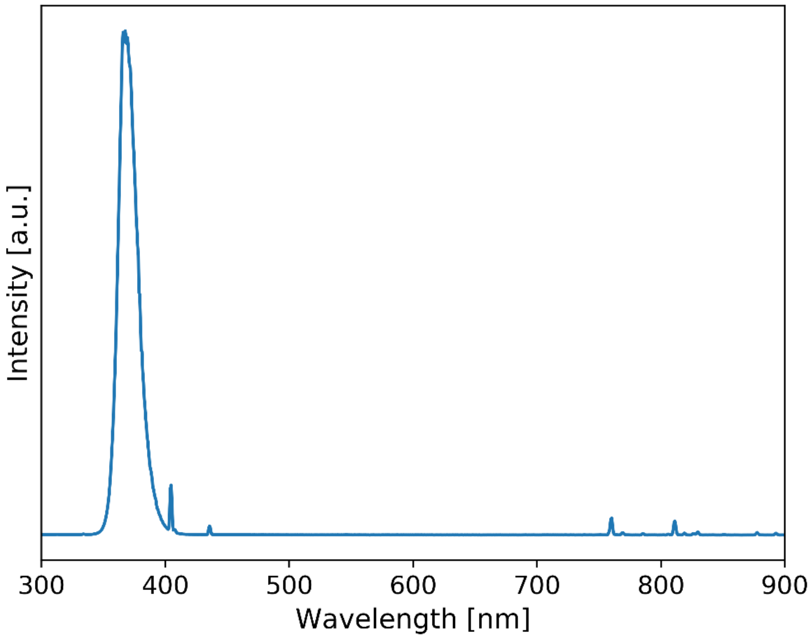

Figure S2. Spectrum of the UV lamp used for the photocatalytic experiments

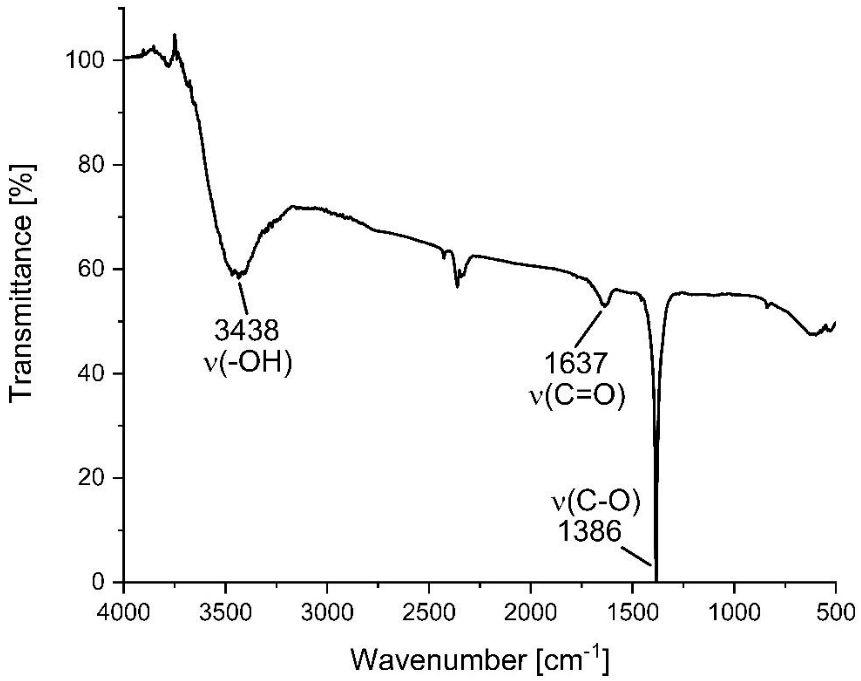

Figure S3. FTIR spectra for sample GO
